# The influence of body fat content and distribution on bone mass in healthy Chinese adults

**DOI:** 10.3389/fmed.2024.1403971

**Published:** 2024-07-23

**Authors:** Bin Chen, Gongwen Liu, Yike Wang, Youjia Xu

**Affiliations:** ^1^Department of Orthopedics, The Second Affiliated Hospital of Soochow University, Suzhou, China; ^2^Department of Orthopedics, Suzhou TCM Hospital Affiliated to Nanjing University of Chinese Medicine, Suzhou, China

**Keywords:** bone mass, fat percentage, osteoporosis, bone mass index, bone mineral density

## Abstract

**Background:**

Previous studies have reported a close relationship between body mass index (BMI) and bone mineral density (BMD). However, the effects of fat on bone mass remain controversial, particularly for fat tissue distribution. The aim of this study was to evaluate the association between regional fat percentage and BMD using a population-based database.

**Methods:**

This study included participants who were referred to the Department of Radio Diagnosis for dual-energy X-ray absorptiometry (DEXA) scan from January 2018 to December 2020. The relationships between BMI and regional fat percentage with BMD were assessed using multiple linear regression and generalized additive models. The risk of low bone mass was determined using logistic regression.

**Results:**

There was a negative relationship between the regional fat percentage and femoral neck BMD (FN BMD) or lumbar spine BMD (LS BMD) in both genders (*p* < 0.05). In females, an inverted U-shaped relationship was observed between regional fat percentage and BMD at both the femoral neck and lumbar spine. The impact of trunk fat percentage on LS BMD was associated with the highest OR of low bone mass in females (OR 3.1, 95% CI 2.6 to 3.7, *p* for trend <0.001), while the impact of abdomen fat percentage on FN BMD was associated with the highest OR of low bone mass in males (OR 2.2, 95% CI 1.8 to 2.7, *p* for trend <0.001).

**Conclusion:**

There was an inverted U-shaped relationship between regional fat percentage and BMD. Excessive regional fat percentage may be harmful to bone health in both genders. To promote bone health, males should restrict their abdomen circumference and avoid abdominal adiposity, while females should control their trunk circumference.

## Introduction

Obesity and osteoporosis are significant global public health concerns, particularly in aging populations. Body mass index (BMI) is a widely used measure to classify adults into categories such as underweight, normal weight, overweight, and obese (1). However, the relationship between BMI and bone mineral density (BMD) remains contentious in the literature (2).

Existing studies suggest that a high BMI may have a protective effect on bone mass, primarily because increased body weight imposes greater mechanical load on bones, thereby stimulating bone formation (3). On the contrary, other studies indicate that excessive BMI may be detrimental to bone health due to metabolic abnormalities associated with obesity that negatively impact bone metabolism. Specifically, adipose tissue is not merely an energy storage organ; it secretes various hormones and cytokines that influence bone metabolism (4, 5).

In examining the impact of body weight on bone mass, the relative importance of fat mass and lean mass has been extensively discussed. For instance, El Hage et al. (6–8) investigated the relative importance of lean and fat mass on BMD in adolescent boys and girls, finding that lean mass is a strong determinant of L1–L4 BMD in boys and that fat mass is a stronger determinant of whole body BMD in girls.

Despite several studies exploring the overall relationship between fat mass and bone mass, the impact of fat distribution on bone mass remains underexplored. Specifically, the effects of regional fat distribution (e.g., trunk fat and abdominal fat) on BMD at different skeletal sites have not been fully elucidated. Lorenzo et al. (9) found that a significant number of male and female subjects could not be classified as obese based solely on their BMI, suggesting that body fat percentage might be a useful indicator. This study aimed to utilize a large-scale population database to investigate the association between regional fat percentage and BMD at the femoral neck (FN) and lumbar spine (LS), thereby advancing the understanding of the complex relationship between adiposity and skeletal health.

## Materials and methods

### Study population

The present retrospective study was conducted at a single study center in China from January 2018 to December 2020. Participants who were referred to the Department of Radio Diagnosis for a DEXA scan were selected. Participants were excluded from the study: (1) History of metabolic bone diseases such as hyperparathyroidism, hyperthyroidism, Cushing’s syndrome, osteomalacia, renal failure, and diabetes mellitus. (2) Those who were taking medications known to influence bone metabolism such as bisphosphonates, estrogen preparations, antiepileptic drugs, corticosteroids, thyroxine, and anticoagulants. (3) Bilateral trunk replacements or previous spinal fusion. (4) non-Han ethnic individuals.

### Clinical measurements

BMI (kg/m2) was calculated as the body weight in kilograms divided by the squared height in meters. BMD was measured at the femoral neck and lumbar spine (L1-L4) as the primary outcome of this study using dual-energy X-ray absorptiometry (DXA, GE-Lunar, Madison, WI, United States). Data on trunk and abdomen fat percentage were extracted from DXA. Based upon the World Health Organization (WHO) classification, a low bone mass (osteopenia or osteoporosis) was defined as a BMD T-score < −1.0 aged above 50 years or a Z-score < −1.0 aged below 50 years. The tests were performed by a trained technician on appropriately calibrated equipment before every session. Densitometers showed stable long-term performances [coefficient of variation (CV) <0.5%] and satisfactory *in vivo* precision (CV 0.8% for lumbar spine; 0.9% for femoral neck).

### Statistical analysis

Continuous variables are presented as the mean ± standard deviation (SD). Comparisons between the males and females were made using independent-samples *t*-test. Linear regression analysis was used to assess the relationships between BMI, trunk, and abdomen fat percentage with BMD in each gender. To obtain greater flexibility in representing the relationships between the dependent variable and predictor variables compared to linear regression, generalized additive models (GAMs) were used to generate graphic representations of the dose–response relations of BMI, trunk, and abdomen fat percentage with BMD in each gender. We performed multiple logistic regression analyses to generate odds ratios (ORs) (95% CI) that compared the odds of low BMD (T-score < −1.0 or Z-score < −1.0) for participants in each of the higher three fat percentage quartile to the odds of the participants in the lowest quartile after adjusting for age, weight, height, and BMI. All analyses were performed using IBM SPSS (version 17, IBM, Chicago, Illinois, United States) and R (version 3.4.3, R Foundation for Statistical Computing, Vienna, Austria), and *p* < 0.05 (two-tailed) was considered statistically significant.

## Results

### General characteristics of the participants

A total of 18,263 participants (8,969 males and 9,294 females) aged 20 to 100 years old were included in the analysis. The demographic details and key clinical data for all participants are shown in Table 1. The mean age of the participants was 48.3 (13.4) years for males and 52.6 (14.3) years for females (*p* < 0.001). Although males had slightly but significantly higher BMI compared to females (24.7 (3.1) vs. 23.0 (3.3) kg/m^2^, *p* < 0.001), they had significantly lower trunk and abdomen fat percentage (18.8 (4.5) vs. 25.6 (5.4) and 26.3 (8.1) vs. 29.2 (8.8), respectively, p < 0.001). Both FN BMD and LS BMD were significantly higher in males than in females (0.95 (0.14) vs. 0.86 (0.15) and 1.13 (0.17) vs. 1.06 (0.20), respectively, *p* < 0.001).

**Table 1:** Descriptive statistics of the study population.

	Female (*n* = 9,294)	Male (*n* = 8,969)	*P* ^a^
	Mean ± SD	Mean ± SD	
Age (y)	52.6 ± 14.3	48.3 ± 13.4	<0.001
Anthropometric data			
Height (cm)	158.6 ± 5.4	170.7 ± 5.8	<0.001
Weight (kg)	57.9 ± 8.7	72.0 ± 10.4	<0.001
BMI (kg/m^2^)	23.0 ± 3.3	24.7 ± 3.1	<0.001
DXA data			
FN BMD (g/cm^2^)	0.86 ± 0.15	0.95 ± 0.14	<0.001
TF (%)	25.6 ± 5.4	18.8 ± 4.5	<0.001
FN T-score (SD)	−0.6 ± 1.3	−0.2 ± 1.1	<0.001
FN BMC (g/cm)	3.9 ± 0.8	5.0 ± 0.9	<0.001
LS BMD (g/cm^2^)	1.06 ± 0.20	1.13 ± 0.17	<0.001
AF (%)	29.2 ± 8.8	26.3 ± 8.1	<0.001
LS T-score (SD)	−0.7 ± 1.7	0.2 ± 1.4	<0.001
LS BMC (g/cm)	29.8 ± 7.1	37.0 ± 7.1	<0.001

Values are mean ± SD unless otherwise stated.

TF (%), trunk fat percentage; AF (%), abdomen fat percentage; FN, femoral neck; LS, lumbar spine; BMD, BMI, body mass index; bone mineral density; BMC, bone mineral content. a Student’s t-test.

### Associations of BMI, trunk, and abdomen fat percentage with BMD in each gender

Regression coefficients from the linear regression models with BMI (Model 1), trunk fat percentage (Model 2), or abdomen fat percentage (Model 3) as the predictor variables are presented in Table 2. In both females and males, BMI serves as a positive predictor of FN BMD and LS BMD (β: 0.20 to 0.32 in females; 0.17 to 0.30 in males, all *p* < 0.001), whereas trunk fat percentage and abdomen fat percentage act as negative predictors of FN BMD and LS BMD (β: −0.04 to −0.18 in females; −0.03 to −0.14 in males, all *p* < 0.05).

**Table 2:** Regression coefficients of models with BMI (kg/m2), TF (%), or AF (%) as the predictor variable for lumbar spine and femoral neck BMD (mg/cm^2^).

	Female		Male	
	Β	GAM Adjusted *R*^2^	β	GAM Adjusted *R*^2^
Model 1	0.32***	0.456	0.30***	0.232
Model 2	−0.07***	0.459	−0.10***	0.238
Model 3	−0.06***	0.458	−0.11***	0.238
LS BMD				
Model 1	0.20***	0.366	0.17***	0.056
Model 2	−0.18***	0.387	−0.14***	0.070
Model 3	−0.04**	0.366	−0.03*	0.056

Model 1: BMI.

Model 2: TF (%), trunk fat percentage.

Model 3: AF (%), abdomen fat percentage.

β: standard regression coefficient.

*P < 0.05; **p < 0.01; ***P < 0.001 compared to the corresponding linear regression model.

Covariates adjusted in both linear regression and GAM included age, weight (for Models 2 and 3 only), and height.

Linear regression analysis with BMD as the dependent variable and BMI (Model 1), TF (%) (Model 2), or AF (%) (Model 3) as the predictor variable.

**Figure 1:**
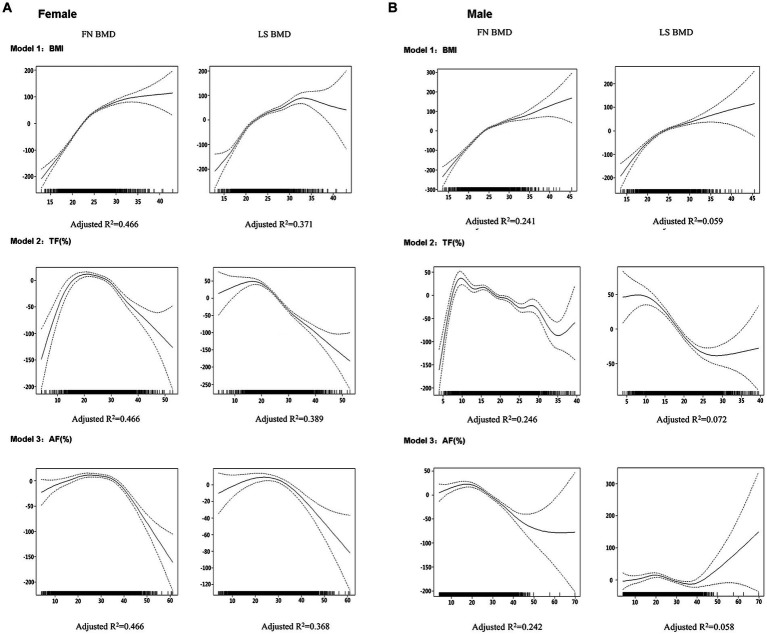
Graphic presentation of the dose–response relationship between BMI (Model 1), TF (%) (Model 2), or AF (%) (Model 3) in males **(A)** and females **(B)** obtained by generalized additive regression models. The models were adjusted for age, weight (for Models 2 and 3 only) and height as covariates. The dotted lines represent 95% confidence intervals. The reference value for the BMD is the value associated with the mean BMI, TF (%), or AF (%) for all participants in each gender. The rug plot along the bottom of each graph depicts each observation.

Figure 1 depicts the dose–response relationships of each BMI (Model 1), trunk fat percentage (Model 2), and abdomen fat percentage (Model 3) with BMD in each gender using the generalized additive models. For BMI, there is a positive relationship with BMD at both femoral neck and lumbar spine across all BMI values in males, whereas in females, BMD increases with BMI until BMI reaches approximately 33 kg/m2, after which there is an apparent decline in BMD at the lumbar spine. For regional fat percentage in males, there appears to be no consistent relationship between BMD and regional fat percentage, whereas, in females, an inverted U-shaped relationship between regional fat percentage and BMD was observed at both femoral neck and lumbar spine. The percentage of variation in the BMD measures explained by the GAM was apparently higher in females compared to males (adjusted *R*^2^: 0.366 to 0.459 vs. 0.056 to 0.238, Table 2), indicating that the relationships were better represented in females by the GAM.

### Associations of trunk and abdomen fat percentage with the odds of low bone mass in each gender

**Figure 2:**
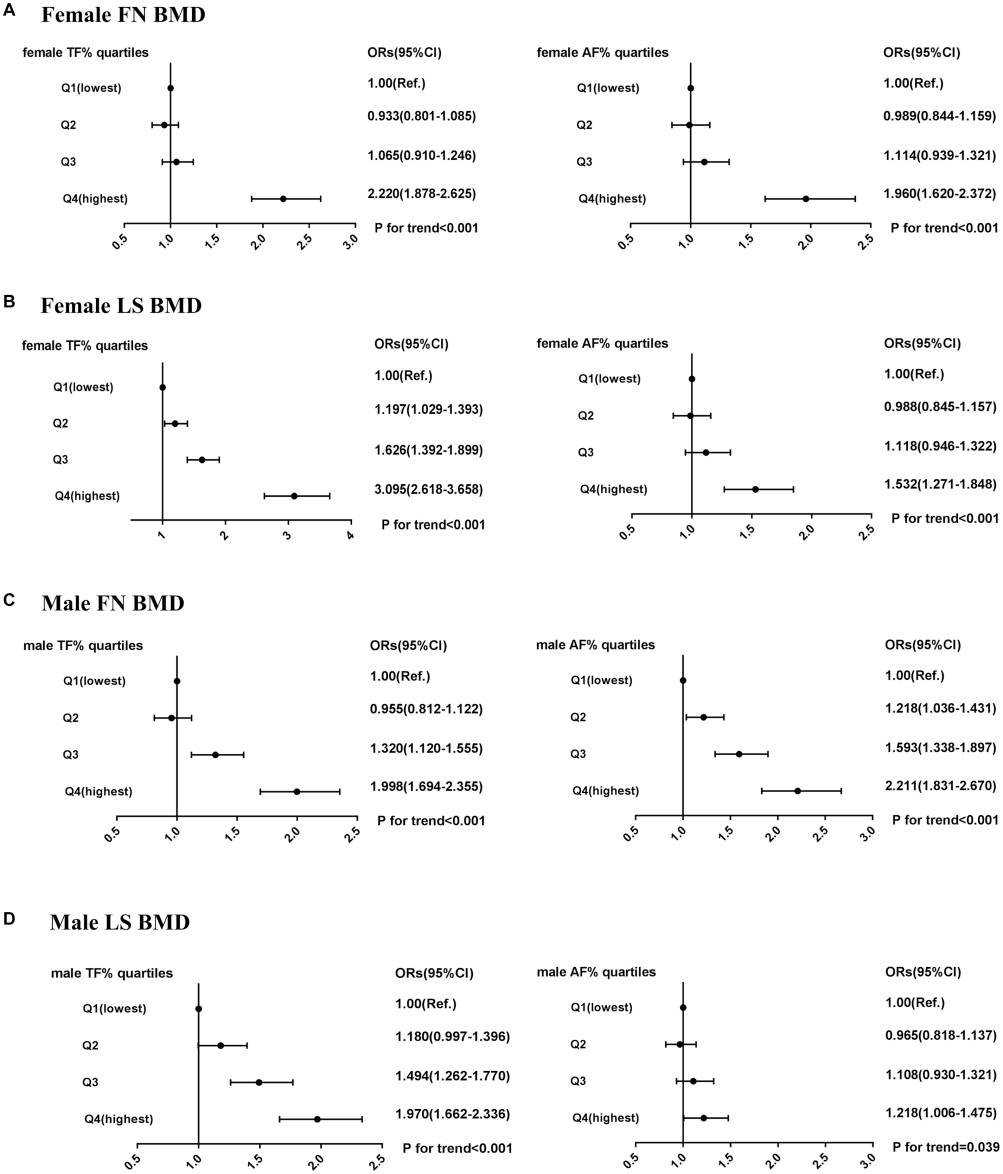
Risk of low bone mass (BMD T-score < −1.0) across quartiles of male TF (%), male AF (%), female TF (%), and female AF (%). **(A)** female FN BMD. **(B)** female LS BMD. **(C)** male FN BMD. **(D)** male LS BMD. ORs (95% CI) were calculated using multivariate logistic regression after adjusting for age, height, and BMI; OR, odds ratio; CI, confidence interval.

As Figure 2 shows, multiple logistic regression analyses showed that the risk of low bone mass was significantly higher in the highest quartile of regional fat percentages compared to that in the lowest quartile in females (ORs ranging from 1.5 to 3.1). The impact of trunk fat percentage on LS BMD was associated with the highest OR of low bone mass in females (OR 3.1, 95% CI 2.6 to 3.7, p for trend <0.001). For males, the risk of low bone mass was significantly higher in the highest quartile of regional fat percentage compared to that in the lowest quartile (ORs ranging from 1.2 to 2.2). The impact of abdomen fat percentage on FN BMD was associated with the highest OR of low bone mass in males (OR 2.2, 95% CI 1.8 to 2.7, p for trend <0.001).

## Discussion

This study has shown that there was a positive relationship with BMD at both femoral neck and lumbar spine across all BMI values in males, whereas in females, BMD increased with BMI until BMI reached approximately 33 kg/m^2^, after which there was an apparent decline in BMD at the lumbar spine. In females, there was an inverted U-shaped relationship between regional fat percentage and BMD at both the femoral neck and lumbar spine. When analyzed by quartiles of regional fat percentage, high trunk fat percentage in females is associated with the highest risk of having low LS BMD, while high abdomen fat percentage in males is associated with the highest risk of having low FN BMD.

The role of body composition on bone health has been extensively investigated, but the results regarding the effect of fat mass on BMD have been controversial. In this study, we found that BMI has shortcomings as a predictor of BMD because it does not separate lean mass from fat mass and does not explore the influence of fat distribution on bone mass. In this study, a positive relationship was observed between BMD at both the femoral neck and lumbar spine across all BMI values in males. However, in females, BMD increased with BMI until reaching approximately 33 kg/m^2^, after which there was an apparent decline in BMD at the lumbar spine. This finding aligns with the results reported by Li (5), who identified an inverted U-shaped association between BMI and lumbar BMD in females, with the point of inflection at approximately 50 kg/m^2^. The variation in the inflection point value may be attributed to racial differences, which could be the result of genetic risk factors, lifestyle, and other factors (10, 11).

In this study, we confirmed that the regional fat percentage was negatively associated with the BMD in males and females using multiple linear regression models. To further investigate the dose–response relationship between the regional fat percentage and the BMD, generalized additive models were performed. In females, the data from our study showed that the relationship between the regional fat percentage and the two-site BMD appeared to be inverted U-shaped, indicating that the effect of the regional fat percentage on the BMD was non-linear. According to these data, we may infer that an increase in regional body fat is weakly protective against bone loss, but this effect becomes detrimental as we move toward morbid obesity. Our results seem consistent with the conclusion from Kim (12) who claimed that overweight may be protective against trunk fractures in Asian adults but not morbid obesity, particularly in women.

Interestingly, through multiple logistic regression models, we found that a high trunk fat percentage in females is associated with the highest risk of having low LS BMD, while a high abdomen fat percentage in males is associated with the highest risk of having low FN BMD. This result was supported by a prospective cohort study from Norway of 23,061 men aged 60 to 79 years, wherein males in the highest tertile of abdomen circumference had a 100% higher risk of trunk fractures than males in the lowest tertile (13). In another cross-sectional study of 1,011 participants aged 50–80 years, it was reported that women who had at least one vertebral deformity had a greater percentage of trunk fat than women without vertebral deformities (14), which was also consistent with our findings above. Altogether, our results indicated that males should control their abdomen circumference and avoid abdominal adiposity, while females should focus on their trunk circumference. Actually, not all fat depots are the same: Site-specific effects, rather than simply total body fat, may be crucial in the assessment of the impact of obesity on the BMD (15).

Several underlying mechanisms have been proposed to elucidate the harmful effect of fat tissue on bone health. At the molecular genetics level, a genome-wide bivariate analysis of Caucasians of European origin identified some suggestive shared genomic regions for both body fat mass and BMD, therefore implying that those two diseases might be influenced by some shared candidate genes or mutual crosstalk between their phenotypes’ gene regulatory networks (16). At the cellular level, adipocytes and osteoblasts have common progenitor cells, mesenchymal stem cells (MSCs). A shift of the cell differentiation of MSCs to adipocytes, rather than osteoblasts, will hinder osteogenesis and will consequently result in bone loss (17). Apart from the causations mentioned above, several adipokines, which are secreted by adipocytes, including adiponectin and leptin, have shown a negative effect on bone metabolism. Serum adiponectin is reported to be inversely correlated with BMD in both males and females by inhibiting osteoblast proliferation and promoting apoptosis, altogether decreasing bone formation levels (18–20). Leptin has a detrimental effect on bone formation mainly via the central nervous system, which appears to be mediated by the decreased production of serotonin in hypothalamic neurons (21, 22).

This study has some limitations. First, it was a retrospective study, which limits the exploration of causality regarding the relationship between high regional fat percentage and BMD. Second, some confounding factors such as sex hormones and adipocytokines were not examined, which could affect the results. Third, information on female menopause was not collected, which could have provided additional insights into the relationship between fat distribution and bone mineral density.

In conclusion, we found that in females, BMD increased with BMI until BMI reached approximately 33 kg/m^2^. Beyond this point, there was an apparent decline in BMD at the lumbar spine. This may be associated with an inverted U-shaped relationship between regional fat percentage and BMD. To promote bone health, males should restrict their abdomen circumference and avoid abdominal adiposity, while females should control their trunk circumference. Excessive regional fat percentage may be harmful to bone health in both genders.

## Data availability statement

The raw data supporting the conclusions of this article will be made available by the authors, without undue reservation.

## Ethics statement

The requirement of ethical approval was waived by the Ethics Committee of the Second Affiliated Hospital of Soochow University for the studies involving humans because this study did not involve patient demographic information and no potentially identifiable images or data were presented in this study. The studies were conducted in accordance with the local legislation and institutional requirements. The ethics committee/institutional review board also waived the requirement of written informed consent for participation from the participants or the participants’ legal guardians/next of kin because this was a retrospective study.

## Author contributions

BC: Conceptualization, Data curation, Formal analysis, Methodology, Project administration, Software, Writing – original draft, Writing – review & editing. GL: Data curation, Investigation, Methodology, Project administration, Software, Validation, Writing – original draft, Writing – review & editing. YW: Supervision, Validation, Writing – original draft, Writing – review & editing. YX: Conceptualization, Funding acquisition, Project administration, Resources, Supervision, Writing – review & editing.
